# Brief Montreal-Toulouse Language Assessment Battery: adaptation and content validity

**DOI:** 10.1186/s41155-020-00157-6

**Published:** 2020-07-30

**Authors:** Raira Fernanda Altmann, Karin Zazo Ortiz, Tainá Rossato Benfica, Eduarda Pinheiro de Oliveira, Karina Carlesso Pagliarin

**Affiliations:** 1 Department of Speech-Language Pathology, Universidade Federal de Santa Maria, Santa Maria/RS, Brazil; 2 Department of Speech-Language Pathology, Universidade Federal de São Paulo, São Paulo, Brazil

**Keywords:** Assessment, Language, Aphasia, Adult, Elderly

## Abstract

**Background:**

Evaluating patients in the acute phase of brain damage allows for the early detection of cognitive and linguistic impairments and the implementation of more effective interventions. However, few cross-cultural instruments are available for the bedside assessment of language abilities. The aim of this study was to develop a brief assessment instrument and evaluate its content validity.

**Methods:**

Stimuli for the new assessment instrument were selected from the M1-Alpha and MTL-BR batteries (Stage 1). Sixty-five images were redesigned and analyzed by non-expert judges (Stage 2). This was followed by the analysis of expert judges (Stage 3), where nine speech pathologists with doctoral training and experience in aphasiology and/or linguistics evaluated the images, words, nonwords, and phrases for inclusion in the instrument. Two pilot studies (Stage 4) were then conducted in order to identify any remaining errors in the instrument and scoring instructions.

**Results:**

Sixty of the 65 figures examined by the judges achieved inter-rater agreement rates of at least 80%. Modifications were suggested to 22 images, which were therefore reanalyzed by the judges, who reached high levels of inter-rater agreement (AC1 = 0.98 [CI = 0.96–1]). New types of stimuli such as nonwords and irregular words were also inserted in the Brief Battery and favorably evaluated by the expert judges. Optional tasks were also developed for specific diagnostic situations. After the correction of errors detected in Stage 4, the final version of the instrument was obtained.

**Conclusion:**

This study confirmed the content validity of the Brief MTL-BR Battery. The method used in this investigation was effective and can be used in future studies to develop brief instruments based on preexisting assessment batteries.

## Introduction

Acquired brain injury due to stroke, traumatic brain injury (TBI), infections, and brain tumors can cause motor impairment, swallowing difficulties and language disorders such as aphasia. Aphasia is most commonly caused by stroke (Martinez, Saborit, Carbonell, & Contreras, 2014; Santiago & Gárate, 2016; Shipley & MacAfee, 2016) and affects approximately one third of stroke sufferers (Koyuncu et al., 2016; Raju & Krishnan, 2015).

Aphasia can be described as a language disorder, in which there is a loss or impairment of the skills of perception, interpretation, and structuring of linguistic elements (Maranhão, Souza, Costa, & Vieira, 2018; Ardila & Rubio-Bruno, 2017). The affected individual may present changes in comprehension and/or oral and/or written expression and difficulties to remember words during a conversation or to name objects (anomy), and the sensory and motor systems (phonoarticulatory apparatus) may be intact (Benson, 1993). Anomy, neologisms, paraphasias, and agrammatisms are some of the linguistic alterations that can be found in aphasic pictures (Ortiz, 2010).

An accurate diagnosis of aphasia is crucial to initiate interventions and improve patient outcomes (Godecke, Hird, Laylor, Rai, & Phillips, 2012; Rohde, Worrall, Godecke, O’Halloran, Farrell, & Massey, 2018). However, it is important to note that language recovery is not a linear process, especially after stroke, where the time since injury is a major contributor to recovery (Kiran & Thompson, 2019).

Many clinical conditions show spontaneous recovery over time, especially in the first 3 months after focal neurological damage (Azuar et al., 2013; El Hachioui et al., 2017). This is attributable to the rapid regeneration of affected tissues, as well as the neurological and functional recovery processes in the subacute phase of stroke (Kiran & Thompson, 2019). This period may see the formation of new synaptic connections and regeneration of damaged pathways (Kiran & Thompson, 2019). Neurogenesis in damaged brain regions may then lead to neural changes which contribute to spontaneous recovery (Kiran & Thompson, 2019). Even though some degree of spontaneous recovery may be expected, it is important to diagnose aphasia while still in hospital in order to allow for the adequate treatment of linguistic and communicative aspects (Johnson et al., 1988; Rohde et al., 2018) and the early implementation of more effective and targeted interventions. As such, language should be evaluated as early as possible in order to guide the rehabilitation process and contribute to prognosis (Nursi et al., 2018).

Extensive aphasia assessment batteries may be too tiring for patients with complex clinical conditions (Casarin et al., 2020). After an acute stroke, for instance, many patients are unable to undergo prolonged evaluations (Marshall & Wright, 2007). In these cases, long assessments may actually constitute a waste of time (El Hachioui et al., 2017). Instruments such as screening tools and brief test batteries which are simpler and easier to administer may be more helpful in diagnosing and mapping the extent of aphasic impairment in inpatient settings (El Hachioui et al., 2017).

Screening instruments can provide information about the language disorder in terms of its impact on comprehension and expression. The results of such an assessment contribute to early rehabilitation interventions, which may lead to greater gains and improve language recovery (Nursi et al., 2018).

Several instruments are currently available to evaluate language in the acute phase of brain injury (Rohde et al., 2018). Internationally available screening instruments originally developed in the English language to screen for language impairment include the following: Acute Aphasia Screening Protocol, AASP (Crary; Haak; Malinsky, 1989); Aphasia Diagnostic Profiles, ADP (Helm-Estabrooks, 1992); Sheffield Screening Test for Acquired Language Disorders, SST (Syder et al., 1993); Frenchay Aphasia Screening Test, FAST (Enderby & Crow, 1996); Mississippi Aphasia Screening Test, MAST (Nakase-Thompson et al., 2005); and Bedside Evaluation Screening Test (2nd edition), BEST-2 (West, Sands, & Ross-Swain, 1998). Other instruments include the Aachen Aphasia Bedside Test, AABT (Biniek, Huber, Glindemann, Willmes, & Klumm, 1992), originally developed in German; the Ullevaal Aphasia Screening Test, UAS (Thommessen, Thoresen, Bautz-Holter, & Laake, 1999), developed in Norwegian; the ScreeLing (Doesborgh et al., 2003), developed in Dutch; and the Language Screening Test, LAST (Flamand-Roze et al., 2011), in French. The BEST-2 and LAST have been translated and adapted to Brazilian Portuguese but are not commercially available.

The majority of English language screening instruments, including the MAST (Nakase-Thompson et al., 2005) and FAST (Enderby & Crow, 1996), detect aphasia using measures of oral and written comprehension, spontaneous speech, repetition, naming, reading, and writing. The same approach is used by the M1-Alpha (Nespoulous et al., 1986), which was the focus of the present study.

All previously mentioned screening instruments are also available in expanded form, as is the M1-Alpha. The latter was developed based on the Montreal-Toulouse Protocol for the Linguistic Assessment of Aphasia, or MT-86, developed by Nespoulous, Joanette, and Lecours (1986). This instrument was translated to Brazilian Portuguese and used for research purposes in the 1980s and 1990s. However, it was never published and was therefore only available to researchers and some clinical practitioners in identifying linguistic behaviors. It is an important instrument in the diagnosis of aphasias, especially in screening hospital environments, which require faster procedures (Ortiz, 1991; Ortiz; Costa, 2011). However, Brazilian studies involving the M1-Alpha identified the need to revise its scoring criteria and revealed issues with some of its linguistic and pictorial stimuli. These included the low recognizability of some pictures, and the absence of tasks considered crucial for assessment and diagnosis (Lecours et al., 1985; Ortiz, Osborn, & Chiari, 1993). In light of these issues, it was suggested that the instrument be revised and updated.

In addition to the M1-Alpha, there is an extended version of the MT-86 protocol known as the MT-86β, which was adapted to Brazilian Portuguese and named *Bateria Montreal-Toulouse de Avaliação da Linguagem* (MTL-BR) (Montreal-Toulouse Language Assessment Battery) (Parente et al., 2016). The MTL-BR has proved fully applicable to the Brazilian population, prompting researchers to consider developing a brief version of the battery similar to the format of the M1-Alpha (Pagliarin et al., 2014; Pagliarin et al., 2014; Pagliarin et al., 2015; Pagliarin et al., 2015; Parente et al., 2016).

The adaptation of neuropsychological instruments is a complex process and must have sufficient psychometric qualities (Gauer, Gomes, & Haase, 2010; Ivanova & Hallowell, 2013; Mcleod & Verdon, 2014; Kirk & Vigeland, 2015; Pacico & Hutz, 2015; Pasquali, 1999). The International Test Commission (ITC, 2017) recommends providing evidence of reliability and validity of the adapted instrument. Therefore, there are four fundamental steps in the adaptation process of a neuropsychological instrument: translation, analysis by non-expert judges, analysis by expert judges, and pilot study (Fonseca et al., 2011).

The content validity is one of the types of validation process of an instrument. This verifies how representative the content selected is for an instrument (Fachel & Camey, 2000; Pasquali, 2009; Oliveira, Sousa, & Maia, 2017). For this, the analysis of expert judges, semantic analysis, and studies with the target population are used. All these raters analyze each item of the instrument taking into account their relevance and clarity (Rubio, Berg-Weger, Tebb, Lee, & Rauch, 2003; Pasquali, 2010; Zamanzadeh et al., 2015; Pernambuco, Espelt, Magalhães, & Lima, 2017).

Few instruments are available for the bedside assessment of linguistic abilities. As such, there is a need for screening tools which can be used to assess patients before hospital discharge so that interventions can be implemented as early as possible. The delayed implementation of speech-language interventions may lead to limited therapeutic progress (Landenberger, Rinaldi, Frison, & Salles, 2017).

In light of the need for earlier detection of language impairments and accurate speech-language diagnoses in patients with acquired brain damage, the aim of this study was to develop a brief instrument for the bedside assessment of linguistic skills in patients with aphasia, based on the MTL-BR Battery and the M1-Alpha. This study will also collect evidence of the content validity of this novel assessment instrument.

## Methods

### Participants and procedures

The study was conducted in four stages, each involving a different set of participants. These included speech pathologists (Stage 1), non-expert judges (Stage 2), expert judges (Stage 3), and a pilot sample (Stage 4). The description and selection criteria for all samples are shown in Table 1.

**Table 1:** Description and selection criteria for participants in each stage of the adaptation and validation of the Brief MTL-BR

Stages	***N***	Selection criteria
**Stage 1** **Evaluation of existing instruments and stimulus selection**	02 aphasia specialists 01 speech pathologist working toward a master’s degree in human communication disorders	Authors of the instrument
**Stage 2** **Non-expert judges**	28 non-expert judges (*N* = 21 female, *N* = 07 male)	Neurologically healthy adults, native speakers of Brazilian Portuguese. At least 5 years of formal education.
**Stage 3** **Expert judges**	09 expert judges (*N* = 5 speech pathologists with doctoral training and experience with language/aphasiology; *N* = 4 speech pathologists with doctoral training and experience with linguistics and phonological acquisition)	Experience in the study of language, aphasiology, and/or linguistics, selected based on clinical and/or research experience.
**Stage 4** **Pilot Study 1** **Pilot Study 2**	07 neurotypical adults (*N* = 4 female, *N* = 3 male) 65 neurotypical adults (*N* = 44 female, *N* = 21 male)	Neurologically healthy adults, right-handed, native speakers of Brazilian Portuguese. At least 5 years of formal education. No signs of depression; no current or prior history of psychoactive substance abuse; no psychiatric, neurological, and/or sensory disorders (uncorrected auditory and/or visual impairment).

The four stages in the adaptation process and validity study are described below.

#### Stage 1. Instrument evaluation and stimulus selection

This stage involved three speech pathologists, including two aphasiologists and one master’s student in Human Communication Disorders. The brief screening battery developed in the present study was based on the adaptation performed by Scliar-Cabral (1983) (M1-Alpha) and the MTL-BR (Parente et al., 2016). The researchers first examined the M1-Alpha (available only to researchers and some clinical practitioners), the MTL-BR, and MTL-BR version B (an alternate form of the MTL-BR developed for psychometric evaluation but not commercially available) in order to select items for the brief assessment instrument and determine whether any of these would need to be redesigned or updated. New stimuli, such as nonwords, were also developed for inclusion in the assessment battery.

Throughout the process, the researchers attempted to ensure that the word structure and sentence length of items in the Brief MTL-BR was similar to those in the M1-Alpha. The administration and scoring instructions, including recommendations for the qualitative analysis of each task, were obtained from the MTL-BR application manual (Parente et al., 2016).

The protocol developed in the present study was named the Brief Montreal-Toulouse Language Assessment Battery (Brief MTL-BR). Since the Brazilian Portuguese equivalent of the MT-86β is referred to as the MTL-BR, the equivalent of the alpha version was named Brief MTL-BR, with the consent of the authors of the original instrument.

#### Stage 2. Assessment by non-expert judges

This stage involved 28 neurologically healthy participants of both genders (21 female and seven male) aged 19 to 61 (M = 29.25, SD = 12.39). Two participants had 5 to 8 years of formal education (7.14%), three had 9 to 11 years (10.71%), and 23 had completed at least 12 years of formal education (82.14%). Participants were recruited from university settings and community centers. For inclusion criteria of non-expert judges, the participants answered the question the Sociodemographic and Health Questionnaire (Fonseca et al., 2012) and underwent the Geriatric Depression Scale (GDS-15) (Yesavage et al., 1983).

In order to verify the representativeness of pictorial stimuli, all 65 images were presented to non-expert judges for analysis. The images were inserted into PowerPoint slides and projected on a screen for each participant. Non-expert judges were asked to perform two tasks: name each image, then match it to one of several descriptive phrases on a sheet of paper. Each participant performed the tasks individually and was asked to suggest any improvements they deemed necessary.

#### Stage 3. Evaluation by expert judges

The sample in this stage consisted of nine speech pathologists with doctoral training and experience in aphasiology and/or linguistics. Participants were selected for the study based on their clinical and/or research experience and invited to participate via an email which also contained a description of the study and of the analysis they would be asked to perform. Five of the judges analyzed 66 drawings in order to classify them as adequate or inadequate considering the representativeness of images of target and distractor stimuli. The other four judges evaluated the words, nonwords, and phrases, classifying them as adequate or inadequate considering psycholinguistic characteristics, such as length, frequency, and mental representativeness. All participants were asked to suggest modifications to the stimuli whenever they deemed necessary. Stimuli classified as inadequate were modified and reevaluated by the same set of judges. After implementation of all suggested improvements, a preliminary version of the Brief MTL-BR was obtained.

#### Stage 4. Pilot study

The pilot study was divided into two parts. In Pilot Study 1, the preliminary version of the instrument was administered to seven participants of both genders (four female and three male), aged 38 to 56 years (M = 45.71, SD = 6.47). Two participants had 5 to 8 years of formal education (29%), four had 9 to 11 years (57%), and one had over 12 years of formal education (14%). The aim of Pilot Study 1 was to identify any problems with the instrument and to estimate the time of administration of the Brief MTL-BR in neurologically healthy participants.

Subsequently, after the implementation of necessary changes, a final version of the Brief MTL-BR was developed. Pilot Study 2 had a similar aim to Pilot Study 1 and involved 65 individuals of both genders (44 female and 21 male) aged 19 to 75 years (M = 42.55, SD = 15.18). A total of 16 individuals had 5 to 8 years of formal education (24.6%), 22 had 9 to 11 years (33.8%), and 27 had at least 12 years of formal education (41.5%). The age of participants in Pilot Study 2 corresponds to the age range of the Brief MTL-BR, though participants were not matched for other characteristics.

Participants in Pilot Studies 1 and 2 were recruited from university settings and community centers. All participants were neurologically healthy, right-handed Brazilian Portuguese speakers, with no current or prior history of psychoactive substance use, and no signs of depression and/or psychiatric or sensory disorders.

In order to screen for exclusion criteria and select participants for Stage 4, prior to completing the Brief MTL-BR, subjects were administered a Sociodemographic and Health Questionnaire (Fonseca et al., 2012) which investigates cultural and communicative experiences, demographic characteristics, handedness, medical history, neurological and motor impairments, as well as the frequency of social interaction and reading and writing activities. Participants also completed the GDS-15 (Yesavage et al., 1983), which was originally developed to screen for signs of depression in elderly populations but is applicable to adults aged 17 or older (Lezak, Howieson, & Loring, 2004). These procedures were conducted in order to screen for exclusion criteria and ensure that participants did not have any health conditions which could interfere with the results of the study. Those selected for participation were then administered the Brief MTL-BR.

### Data Analysis

Each stage of the study involved a different set of statistical procedures. Data from Stages 1 and 4 were analyzed using descriptive methods. Stage 2 involved the calculation of simple percent agreement between raters. Items with a minimum of 80% agreement were maintained in the instrument (Fagundes, 1985).

In Stage 3, the Content Validity Ratio (CVR) (Lawshe, 1975) of each item was analyzed. The CVR was obtained using the following formula: CVR = (ne − *N*/2)(N/2), where ne corresponds to the number of positive ratings for a given item, and *N* represents the total number of raters. The minimum acceptable CVR depends on the number of raters. For a study with five raters, the minimum acceptable value is 0.99 (Pacico & Hutz, 2015). After the CVR analysis, inter-rater agreement was evaluated using Gwet’s first-order agreement coefficient (AC1) (Gwet, 2008).

### Ethical aspects

This study was conducted as part of a research project approved by the Federal University of Santa Maria Ethics Board under protocol number 2.170.519. All participants provided written informed consent prior to entering the study, as recommended by National Health Council Resolution 466/12. Authorization for this study was also obtained from the authors of the original M1-Alpha, as provided by the ITC guidelines (2017).

## Results

In Stage 1, the analysis of items from the extended MTL-BR, the M1-Alpha, and the MTL-BR version B revealed the need to substitute or adapt some of the stimuli from these instruments. A total of 120 images were selected for the Brief MTL-BR Brief, including 25 from the M1-Alpha, 52 from the MTL-BR version B, and 43 newly developed items. Pictographic stimuli have fine lines drawn in black and white. A total of 14 words (11 from the M1-Alpha and 3 newly developed) were also selected, as were five nonwords and five sentences (one from the M1-Alpha and four newly developed). Two tasks which are not in the M1-Alpha protocol were also added to the Brief MTL-BR. These were the automatic speech and nonverbal praxis tasks, both from the MTL-BR Battery.

Sixty-five images were redrawn and reanalyzed by the non-expert judges. The results of this procedure revealed 100% agreement on 33 images, 96% agreement on 19 images, 93% agreement in six images, and 89% agreement on two images. Five images failed to achieve the 80% agreement threshold and were therefore modified. These results led to the substitution of six images and the addition of one image to two response cards.

After these changes were made, the five specialist judges were given 66 images for analysis (Stage 3). A third of these items had unsatisfactory ratings, with a CVR of − 0.20 to 0.60. These were therefore redesigned. The remaining 44 items had a CVR = 1 and remained in the instrument. The 22 redesigned items were then reexamined by the expert judges. Three items obtained a CVR of 0.6 while the remaining 19 had a CVR = 1. After careful analysis by the authors of the present study, it was decided that the three items with a low CVR would remain in the instrument but as distractors rather than target stimuli. Additionally, Gwet’s agreement coefficient suggested near perfect inter-rater reliability (AC1 = 0.98; CI = 0.96–1).

The four remaining specialist judges analyzed 14 words, five nonwords, and four sentences for inclusion in the Brief MTL-BR (Stage 3). Eight words had 100% inter-rater agreement, while six had 75%. Four nonwords had 100% agreement while one had 75%. Lastly, two of the sentences had 100% agreement, one reached 75%, and the other, 50% agreement. After analyzing the suggestions made by expert judges, the authors of this study opted to make no modifications to the items, since this would have detracted from the purpose of the instrument. One suggestion, for instance, pertained to the sentence “The apples are green” in the dictation task. The judges suggested the word “apples” (in Portuguese, “maçãs”) be replaced by a regularly spelled word. However, assessing the spelling of irregular words is one of the main goals of the task. Additionally, Gwet’s agreement coefficient suggested satisfactory inter-rater reliability for this item (AC1 = 0.74, CI = 0.57–0.90).

These procedures were followed by Stage 4, which began with Pilot Study 1. Seven participants were administered the preliminary version of the Brief MTL-BR. The mean duration of administration was 8 min (SD = 1.25). During the pilot study, some errors were detected in the instrument, including punctuation errors in two tasks (guided interview and oral comprehension) and an inadequate stimulus in the written comprehension task. After the stimulus in question was redesigned and the punctuation errors were corrected, the final version of the Brief MTL-BR was obtained. This instrument did not require any additional modifications and was therefore used in Pilot Study 2. The mean time of administration of the final version of the Brief MTL-BR in healthy adults was 11 min (SD = 4.00).

The Brief MTL-BR was therefore composed of the following tasks: directed interview, oral comprehension, written comprehension, copy, writing to dictation, reading aloud, oral naming, automatic speech, and non-verbal praxis. The directed interview task in the Brief MTL-BR is identical to the corresponding task in the M1-Alpha, save for minor modifications, such as the inclusion of the terms “in treatment/sick” in the question, “How long have you been in the hospital?” In the oral comprehension task (words, simple, and complex phrases), four of the stimuli obtained from the M1-Alpha were updated, and five new response cards were developed. Two response cards from the MTL-BR version B were also included in the Brief MTL-BR. The written comprehension task (words, simple, and complex phrases) was also modified. Three new cards were developed, while seven were reused from the MTL-BR version B. Only one response card form the M1-Alpha was updated and reused.

The copying task involved the same sentence as the M1-Alpha. In the writing to dictation task, all words were modified, and nonwords were included. The sentence in this task was also changed but retained the same structure and number of words as the sentence in the corresponding task of the M1-Alpha. The copy and writing to dictation tasks were marked as optional in the MTL-BR Brief, since the instrument is intended for bedside assessment, and some patients in this situation may be unable to complete these items.

The repetition test retained five of the eight words in the M1-Alpha protocol. Of the remaining three words, one underwent phonological modification (e.g., “cat”/“gato” was substituted for “duck”/“pato”), and two were replaced with pseudowords. A new sentence was also created for this task. In the reading aloud task, two words were replaced with pseudowords. A new phrase was also developed.

In the oral naming task, four stimuli (one noun and three verbs) were reused from the MTL-BR version B, three nouns were selected from the M1-Alpha, and five new stimuli were developed. One of the newly developed items was in the same semantic category as an item in the M1-Alpha (e.g., “nose” was substituted for “ear”). Automatic speech and nonverbal praxis tasks were also included in the Brief MTL-BR. Table 2 shows tasks, objectives, and number of items used on the instrument.

**Table 2:** Description of Brief MTL-BR tasks

Tasks	Objective	Items
Directed interview	Assess oral expression and comprehension.	Contains 9 open questions.
Auditory comprehension	Measures spoken word and sentence comprehension, by asking participants to identify a picture corresponding to the named stimulus.	The task contains 11 items, consisting of 5 words, 3 simple sentences, and 3 complex sentences. Words items are presented one at a time and accompanied by six stimuli each, namely, a correct target image, a phonemic distractor, a semantic distractor, a visual distractor, and two neutral images.
Written comprehension	Assesses the ability to identify images corresponding to written words and sentences.	The task contains 11 items and includes 5 words, 3 simple sentences, and 3 complex sentences. The words stimuli are presented one at a time and accompanied by 6 pictures each, namely, a correct target image, a phonemic distractor, a semantic distractor, a visual distractor, and two neutral images.
Sentence copying	Assesses the ability to recognize and reproduce letters.	The task is composed of one 4-word sentence.
Writing to dictation	Assesses the ability to comprehend auditory input and find the corresponding written representation.	The task contains 4 words (regular, irregular, and pseudowords) and 1 sentence.
Repetition	Assesses the ability to verbally reproduce spoken words and sentences.	The task contains 6 words (regular, irregular, and pseudowords) and 1 sentence.
Reading	Investigates the ability to recognize letters and respond with the corresponding phoneme.	The task contains 8 words (regular, irregular, and pseudowords) and 1 sentence.
Naming	Assesses the ability to name pictures of nouns and verbs.	A total of 12 images are individually presented, depicting 9 nouns and 3 verbs.
Automatic speech	Assesses the ability to produce automatic series.	Numbers and music Happy Birthday,
Nonverbal praxis	Assesses the ability to produce isolated gestures and movement sequences involving the face and tongue, following instructions by the examiner.	The task is composed of 6 items.

## Discussion

To detect linguistic changes resulting from neurological damage, language assessment is essential (Kalbe, Reinhold, Brand, Markowitsch, & Kessler, 2005). If this is carried out still at the bedside, an initial overview of the deficits presented can be obtained, favoring that the beginning of the speech therapy intervention is early (Kiran & Thompson, 2019; Salter, Jutai, Foley, Hellings & Teasell, 2006; Sampaio & Moreira, 2016). For assessments to occur during hospitalization, it is necessary that the test be performed quickly and effectively (Seniów, Litwin, & Leśniak, 2009).

Due to the existing limitation of language tests available for aphasia that present psychometric evidence, it is necessary to adapt instruments to fill this gap in clinical practice and research. It also emphasized the importance of tests guarantee that materials are culturally and linguistically sensitive (Ivanova & Hallowell, 2013).

When developing an instrument based on an existing assessment tool, it is important to ensure that it remains similar in content to the original instrument, even after implementing any necessary modifications (Borsa & Seize, 2017). Additionally, the items should be approved by the authors of the original instrument (Astepe & Köleli, 2019), as was the case in the present study. The stimuli in the Brief MTL-BR were selected based on existing instruments such as the MTL-BR and the M1-Alpha, as well as the MTL-BR version B. The resulting instrument fulfilled the same purpose as the M1-Alpha protocol, using updated and redesigned stimuli.

Additionally, brief measures of automatic behavior were included in the Brief MTL-BR. A similar procedure was followed in the MTL-BR, since these abilities are often preserved in severe aphasia and must therefore be examined (Vendrell, 2001). The Brief MTL-BR evaluates automatic verbal behavior through counting and singing “Happy Birthday.” In addition to these items, the MTL-BR also requests that patients name the days of the week. The importance of evaluating automatic speech was made evident in studies involving instruments such as the MAST (Nakase-Thompson et al., 2005), LAST (Flamand-Roze et al., 2011), and MTL-BR (Parente et al., 2016).

Items were also added to the instrument in order to evaluate nonverbal praxis, including the ability to perform isolated gestures and sequences of tongue and face movements (Parente et al., 2016), which may provide an indication of impaired nonverbal motor planning in addition to language disorders (Bonini & Radanovic, 2015; Rouse, 2020). The number of sentences in the repetition and reading aloud tasks was also reduced to facilitate screening. Instruments such as the Mini Mental State Examination (Chaves & Izquierdo, 1992), which are also used for screening purposes, are composed of short tasks, which are easy to administer and provide a cognitive profile of the patient. Additionally, pseudowords were included in the reading aloud, writing to dictation, and repetition tasks, in order to evaluate the perilexical or phonological reading route. These items follow the same structure of real words in Brazilian Portuguese. Irregular words were also included in the reading and dictation tasks in order to evaluate the lexical reading route. This type of stimulus serves a similar purpose in other instruments such as the MTL-BR (Parente et al., 2016) and the Boston Diagnostic Aphasia Examination (BDAE) (Goodglass & Kaplan, 1983).

Content validity is crucial for the adaptation and development of assessment instruments, as it provides information about the relevance, clarity, and representativeness of each item (Sireci, 1998; Oliveira, Sousa, & Maia, 2017). Therefore, after items are selected for a particular instrument, it is important for their semantic properties to be assessed by the target population as well as expert judges in order to collect evidence of content validity (Pasquali, 2010; Borsa & Seize, 2017). In the present study, the first of these procedures was referred to as “evaluation by non-expert judges” (Stage 2) and confirmed the intelligibility of the images in the instrument.

As instructed by the ITC’s guidelines (2017), expert judges must consider linguistic, cultural, and psychological differences in the intended population of an instrument. In this study, the analysis by expert judges (Stage 3) showed that the stimuli selected or developed for the instrument were adequate. The expert judges were asked to determine whether the stimuli were representative and consistent with the goals of the instrument (Mohajan, 2017; Zamanzadeh et al., 2015). The CVR was used to measure inter-rater agreement and determine the extent to which an item was considered essential for the test, as has been done in previous studies (Al-Thalaya et al., 2017; Bonini; Keske-Soares, 2018). Previous instrument adaptation studies which used Gwet’s first-order agreement coefficient (AC1) found that values over 0.70, such as those obtained in the present study, are indicative of satisfactory agreement between raters (Bukenya et al., 2017; Erivan et al., 2019). The main suggestion offered by judges who analyzed the words, pseudowords, and phrases for the Brief MTL-BR was the replacement of irregular by regular words. However, the authors of the present study chose not to accept this suggestion, since they believed irregular words should remain in the instrument to allow for an assessment of lexical strategies for reading and writing, both of which are important aspects of linguistic processing (Pinheiro & Rothe-Neves, 2001).

The pilot study (Stage 4) was crucial to provide an estimate of the time of administration and detect any errors in the instrument. According to the literature, using an instrument in a realistic situation often allows for the identification of issues which may have gone unnoticed in other steps of the study (Salles et al., 2011; Bailer, Tomitch, & D’Ely, 2011). Additionally, the pilot study helps evaluate the comprehensibility of test items and instructions, detect insufficiently sensitive tasks, and familiarize the examiners with scoring methods (Salles et al., 2011). According to the ITC guidelines (2017), confirmatory evidence about the psychometric quality of the adapted instrument should be obtained. The pilot study is a way of verifying the functioning of the test items and instructions and revising them when necessary.

The duration of administration of the Brief MTL-BR (11 min) is similar to that of instruments such as the UAS (Thommessen et al., 1999). As such, the Brief MTL-BR can be considered a brief assessment instrument suitable for inpatient screening, like the M1-Alpha (Ortiz, Osborn, & Chiari, 1993). However, this does not mean that the administration in acute aphasia sample will have the same duration. Further studies considering that will be conduct.

Like screening instruments in the English language such as the MAST (Nakase-Thompson et al., 2005), which is internationally recognized as a major screening tool for aphasia (Salter et al., 2006), the Brief MTL-BR includes measures of both receptive and expressive language. However, in addition to evaluating linguistic abilities, the Brief MTL-BR also allows for an assessment of non-verbal praxis, unlike the MAST or other language screening tools.

## Conclusion

The present study provided a detailed account of the adaptation of the Brief MTL-BR, which confirmed its content validity and applicability to adult and elderly individuals. The tasks selected for this instrument from the M1-Alpha and MTL-BR (Parente et al., 2016) capture the most significant language impairments in patients with left hemisphere damage. However, there is still a need for further studies involving the clinical population which is the target of this assessment battery. Therefore, other psychometric sources should also be researched such as test-criterion, convergent validity, factor analysis, and consequences of testing.

Given the scarcity of language screening instruments for patients with left hemisphere damage in Brazilian Portuguese, the Brief MTL-BR constitutes an important contribution to the current literature. The present findings provided strong evidence of the content validity of the MTL-BR Brief. Further studies are required to investigate its reliability, as well as its construct and criterion validity.

## Data Availability

All data generated and analyzed during this study will be treated with total confidentiality. The dataset supporting the conclusions of this article is available by request to the authors.

## Abbreviations

AABT: Aachen Aphasia Bedside Test
AASP: Acute Aphasia Screening Protocol
ADP: Aphasia Diagnostic Profiles
BEST: Bedside Evaluation Screening Test
CVR: Content Validity Ratio
FAST: Frenchay Aphasia Screening Test
GDS-15: Geriatric Depression Scale
ITC: International Test Commission
LAST: Language Screening Test
MAST: Mississippi Aphasia Screening Test
MTL-BR: Montreal-Toulouse Language Assessment Battery
SST: Sheffield Screening Test for Acquired Language Disorders
TBI: Traumatic brain injury
UAS: Ullevaal Aphasia Screening Test
