# Correction to: Brief Montreal-Toulouse Language Assessment Battery: adaptation and content validity

**DOI:** 10.1186/s41155-020-00162-9

**Published:** 2020-09-30

**Authors:** Raira Fernanda Altmann, Karin Zazo Ortiz, Tainá Rossato Benfica, Eduarda Pinheiro Oliveira, Karina Carlesso Pagliarin

**Affiliations:** 1 Department of Speech-Language Pathology, Universidade Federal de Santa Maria, Santa Maria/RS, Brazil; 2 Department of Speech-Language Pathology, Universidade Federal de São Paulo, São Paulo, Brazil

**Correction to: Psicol Refl Crít 33, 18 (2020)**

**https://doi.org/10.1186/s41155-020-00157-6**

Following publication of the original article (Altmann et al., 2020), the authors identified an error in the author name of Eduarda Pinheiro Oliveira.

The incorrect author name is: Eduarda Pinheiro de Oliveira

The correct author name is: Eduarda Pinheiro Oliveira

The author group has been updated above.
